# Ex-vivo quantitative susceptibility mapping of human brain hemispheres

**DOI:** 10.1371/journal.pone.0188395

**Published:** 2017-12-20

**Authors:** Arnold M. Evia, Aikaterini Kotrotsou, Ashish A. Tamhane, Robert J. Dawe, Alifiya Kapasi, Sue E. Leurgans, Julie A. Schneider, David A. Bennett, Konstantinos Arfanakis

**Affiliations:** 1 Department of Biomedical Engineering, Illinois Institute of Technology, Chicago, IL, United States of America; 2 Rush Alzheimer's Disease Center, Rush University Medical Center, Chicago, IL, United States of America; 3 Department of Diagnostic Radiology, Rush University Medical Center, Chicago, IL, United States of America; 4 Department of Pathology, Rush University Medical Center, Chicago, IL, United States of America; 5 Department of Neurological Sciences, Rush University Medical Center, Chicago, IL, United States of America; Pennsylvania State University College of Medicine, UNITED STATES

## Abstract

Ex-vivo brain quantitative susceptibility mapping (QSM) allows investigation of brain characteristics at essentially the same point in time as histopathologic examination, and therefore has the potential to become an important tool for determining the role of QSM as a diagnostic and monitoring tool of age-related neuropathologies. In order to be able to translate the ex-vivo QSM findings to in-vivo, it is crucial to understand the effects of death and chemical fixation on brain magnetic susceptibility measurements collected ex-vivo. Thus, the objective of this work was twofold: a) to assess the behavior of magnetic susceptibility in both gray and white matter of human brain hemispheres as a function of time postmortem, and b) to establish the relationship between in-vivo and ex-vivo gray matter susceptibility measurements on the same hemispheres. Five brain hemispheres from community-dwelling older adults were imaged ex-vivo with QSM on a weekly basis for six weeks postmortem, and the longitudinal behavior of ex-vivo magnetic susceptibility in both gray and white matter was assessed. The relationship between in-vivo and ex-vivo gray matter susceptibility measurements was investigated using QSM data from eleven older adults imaged both antemortem and postmortem. No systematic change in ex-vivo magnetic susceptibility of gray or white matter was observed over time postmortem. Additionally, it was demonstrated that, gray matter magnetic susceptibility measured ex-vivo may be well modeled as a linear function of susceptibility measured in-vivo. In conclusion, magnetic susceptibility in gray and white matter measured ex-vivo with QSM does not systematically change in the first six weeks after death. This information is important for future cross-sectional ex-vivo QSM studies of hemispheres imaged at different postmortem intervals. Furthermore, the linear relationship between in-vivo and ex-vivo gray matter magnetic susceptibility suggests that ex-vivo QSM captures information linked to antemortem gray matter magnetic susceptibility, which is important for translation of ex-vivo QSM findings to in-vivo.

## Introduction

Quantitative susceptibility mapping (QSM) is a recently developed magnetic resonance imaging (MRI) technique that has shown promise in investigations of age-related diseases [1, 2]. Magnetic susceptibility of tissue is derived from its molecular and cellular composition, and may allow for the differentiation of healthy and diseased tissue [3–6]. The application of QSM in aging is an active field of research with a number of studies suggesting that MRI is sensitive to magnetic susceptibility changes in the brain of older persons with a clinical diagnosis of Alzheimer’s [7–9], Huntington’s [10–12], or Parkinson’s disease [13–18]. Combining QSM with postmortem assessments of the neuropathologies responsible for these susceptibility changes is essential for determining the role of QSM as a diagnostic and monitoring tool in age-related diseases [19, 20].

Combining in-vivo brain MRI and postmortem neuropathologic evaluation in the same older adults has important inherent limitations. First, the MRI data most proximal to autopsy are typically collected years prior to death, and additional pathology may develop during that period. This leads to an underestimation of the pathology-related brain changes that can be detected with MRI. Furthermore, studies of aging using in-vivo MRI are biased towards less frail individuals who are more likely to volunteer for a scan. In contrast, ex-vivo MRI addresses the above limitations by providing an assessment of brain characteristics for the same condition of the brain as that assessed histologically [21, 22], and by allowing brain imaging of both well-functioning as well as frail older adults. However, to realize the full potential of combining ex-vivo MRI and neuropathologic evaluation, one must be able to translate the ex-vivo MRI findings to in-vivo. Therefore, it is crucial to understand the effects of death and chemical fixation on brain MRI data collected ex-vivo. Such investigations have been conducted for ex-vivo measurements of regional brain volumes [23], T_1_ [24], and T_2_ [25] values. However, the effects of death and chemical fixation on the behavior of magnetic susceptibility of human brain hemispheres over time postmortem have not been systematically investigated. This information is important for future cross-sectional ex-vivo QSM studies on brain hemispheres imaged at different postmortem intervals. Also, the relationship between magnetic susceptibility values measured in-vivo and ex-vivo is not known. This is essential for translating any ex-vivo QSM findings to in-vivo.

The purpose of this work was twofold: a) to assess the behavior of magnetic susceptibility in both gray and white matter of human brain hemispheres as a function of time postmortem, and b) to establish the relationship between in-vivo and ex-vivo gray matter susceptibility measurements on the same hemispheres. To assess the longitudinal behavior of ex-vivo magnetic susceptibility in both gray and white matter, QSM data on five human brain hemispheres imaged on a weekly basis for six weeks postmortem, were analyzed. The relationship between in-vivo and ex-vivo gray matter susceptibility measurements was investigated using QSM data from eleven participants imaged both antemortem and postmortem.

## Methods

### Participants

Seventeen participants were recruited from the Rush Memory and Aging Project (MAP) and Religious Orders Study (ROS), two longitudinal epidemiologic clinical-pathologic cohort studies of aging [26, 27]. All participants provided written informed consent, underwent biennial MR imaging, and signed an anatomical gift act. The studies were approved by the Institutional Review Board of Rush University Medical Center.

### Tissue handling protocol

**Fig 1:**
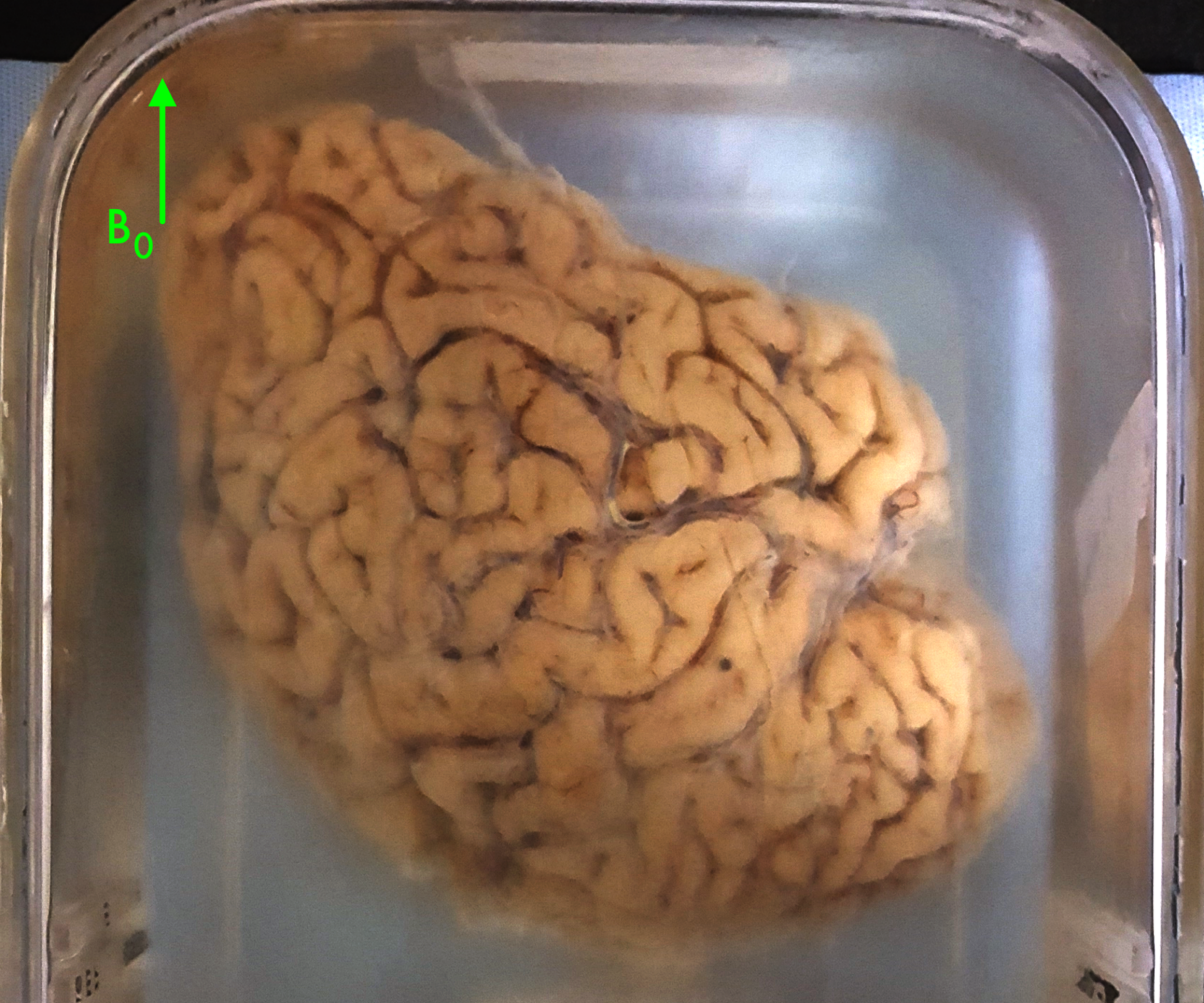
Postmortem brain hemisphere in imaging container. Example of a participant’s brain hemisphere submerged in formaldehyde solution.

Following a participant's death, an autopsy technician extracted the brain and separated the cerebrum from the cerebellum and brainstem. The cerebrum was then bisected into left and right hemispheres, and the hemisphere with more extensive visible gross abnormalities was chosen for ex-vivo MRI, while the contralateral hemisphere was frozen and stored. The chosen hemisphere was immersed in phosphate-buffered 4% formaldehyde solution (prepared from paraformaldehyde) and stored at 4°C within 30 minutes after extraction. Prior to ex-vivo MRI, the hemisphere was transferred to a clear plastic container filled with formaldehyde solution, was positioned with its medial aspect towards the bottom of the container, and was allowed to return to room temperature (Fig 1). Ex-vivo MRI was conducted as described in the next section. Histopathologic examination was performed within 2 weeks after ex-vivo MRI, by a board-certified neuropathologist. Additional details can be found in [23].

### MRI data acquisition

Three datasets were used in this work. Dataset 1 was used to assess the behavior of ex-vivo human brain magnetic susceptibility over time postmortem. Datasets 2 and 3 were used to establish the relationship between magnetic susceptibility measurements performed in-vivo and ex-vivo.

#### Dataset 1

Ex-vivo MRI data were collected on a weekly basis for six weeks postmortem on cerebral hemispheres from five participants (Table 1) (all participants died of natural causes) using a 3T Philips MRI scanner (Best, The Netherlands) with an 8-channel head coil and the following protocol: a) 3D gradient multi-echo sequence with 10 echoes, TE_1_/ΔTE/TR = 4.6/4.63/49.5 ms, flip angle = 22 degrees, 65 sagittal slices, slice thickness = 1 mm, field of view (FOV) = 16 cm x 16 cm, acquisition matrix = 160x160, image matrix = 160x160, scan time = 8m 29s, and b) multi-echo spin-echo sequence with 5 echoes, TE_1_/ΔTE/TR = 16.5/16.5/4055 ms, 42 sagittal slices, slice thickness = 1.5 mm, FOV = 16 cm x 16 cm, acquisition matrix = 260x258, image matrix = 288x288, repetitions = 2, scan time = 35 m. Cerebral hemispheres were positioned in the main magnetic field, B_0_, as shown in Fig 1.

**Table 1:** Dataset 1 characteristics.

Participant	A	B	C	D	E
Age at death (years)	93.0	96.4	89.9	86.7	99.4
Postmortem interval to immersion in fixative (hours)	10.5	5.9	7.7	7.7	8.3
Hemisphere side	Right	Right	Right	Right	Right
Sex	F	F	F	F	F
Likelihood of Alzheimer's based on NIA-Reagan criteria	Intermediate	Intermediate	Intermediate	High	Low
Lewy Bodies	None	None	None	None	Present
Cerebral Amyloid Angiopathy	None	Moderate	Mild	Severe	None
Hippocampal Sclerosis	Present	None	None	None	None
Gross infarcts	None	None	None	None	Present
Microinfarcts	None	Present	Present	Present	Present

Demographic and imaging characteristics, and pathologic diagnosis for individuals in Dataset 1. The methods used for pathologic diagnosis have been described elsewhere [19].

#### Dataset 2

Both in-vivo and ex-vivo MRI data were collected on eleven participants (Table 2) (all participants died of natural causes). All participants were scanned in-vivo between 0.3 to 3.6 years antemortem using a 1.5T General Electric MRI Scanner (Waukesha, WI) and the following protocol: 2D gradient-echo sequence with 2 echoes and flow compensation, TE_1_/TE_2_/TR = 8/17.1/2000ms, flip angle = 80 degrees, 36 axial slices, slice thickness = 3 mm, FOV = 24 cm x 24 cm, acquisition matrix = 128 x 128, scan time = 4m 24s. Hemispheres from the same participants were also imaged approximately 30 days postmortem (Table 2), using the same scanner and MRI pulse sequences as described in Dataset 1.

**Table 2:** Dataset 2 characteristics.

Participant	F	G	H	I	J	K	L	M	N	O	P
Age at death (years)	91.3	93.6	88.2	89.5	92.6	89.4	86.5	93.4	88.9	85.8	92.1
Antemortem interval (years)	1.2	2.1	2.3	0.8	1.1	0.3	3.0	3.6	3.1	1.5	2.2
Postmortem interval to immersion in fixative (hours)	6.5	6.7	4.8	5.9	6.1	7.2	5.7	5.9	10.2	6.3	6.7
Postmortem interval to imaging (days)	31	31	31	32	29	33	35	36	34	29	34
Hemisphere side	Left	Right	Right	Right	Right	Right	Right	Right	Right	Left	Left
Sex	M	M	F	M	M	F	F	F	M	F	F
Likelihood of Alzheimer's based on NIA-Reagan criteria	Low	Low	High	Low	Low	Low	Intermed.	Low	Intermed.	Intermed.	Intermed.
Lewy Bodies	None	None	None	Present	None	None	None	None	None	None	Present
Cerebral Amyloid Angiopathy	None	Mild	None	Mod.	Mild	Mild	None	Mild	Mild	Mod.	Mild
Hippocampal Sclerosis	None	None	None	None	None	None	None	None	None	None	None
Gross infarcts	None	None	Present	None	None	None	None	None	None	Present	None
Microinfarcts	None	Present	Present	None	None	None	None	Present	None	Present	None

Demographic and imaging characteristics, and pathologic diagnosis for individuals in Dataset 2. The methods used for pathologic diagnosis have been described elsewhere [19].

#### Dataset 3

Since in Dataset 2, in-vivo data were collected on a 1.5T scanner near the participants’ homes, while ex-vivo data were collected on a 3T scanner near the tissue bank, one brain hemisphere was imaged ex-vivo using both protocols (on the 1.5T and 3T scanners) to investigate protocol-related effects on the relationship between in-vivo and ex-vivo magnetic susceptibility measurements. The two imaging sessions were performed on the same day.

### Quantitative susceptibility mapping

#### Ex-vivo preprocessing

Phase maps were generated for all echoes of the 3D gradient multi-echo acquisition from each hemisphere by combining the corresponding real and imaginary data. Voxels affected by air-tissue interfaces (e.g. due to air trapped inside the ventricles or on the surface of the hemisphere) were masked out.

#### Ex-vivo background field removal

A total field map was produced for each brain hemisphere by voxel-wise linear fitting of the unwrapped phase values (PRELUDE) [28] of the second to fifth echoes. The background field was removed from the total field map using Regularization Enabled Sophisticated Harmonic Artifact Reduction for Phase data (RESHARP) [29]. The regularization parameter that controls the tolerance of large local field changes was determined for each hemisphere with the L-curve method [30].

#### Ex-vivo dipole inversion

A magnetic susceptibility map was created for each hemisphere using the L1-regularized Morphology Enabled Dipole Inversion (MEDI) technique [31]. The regularization parameter that affects the smoothness of the susceptibility map in MEDI was empirically determined as 400 based on the data from two participants, and was applied to all participants. Streaking artifacts in the susceptibility map were suppressed by imposing consistency on the cone data (CCD) [32]. The edge map for both MEDI and CCD was derived from the participant’s R_2_* map, which was generated from the slope of the voxel-wise linear fit of the natural logarithm of the signals from the second to fifth echoes. The reference susceptibility value was chosen as the mean susceptibility of the surrounding formaldehyde solution.

#### In-vivo QSM processing

In-vivo magnetic susceptibility maps were generated with similar methodology as that described for ex-vivo data, but due to differences between the in-vivo and ex-vivo acquisition protocols, there were necessary modifications in processing approaches. More specifically, for local field mapping, a 2D SHARP method with a regularization parameter of 0.20 (determined based on the criteria of Kaaouana et al. [33]) was used on the second echo. R_2_* maps were generated based on two echoes. The value of the regularization parameter for MEDI was determined with the procedure described above as 800. Finally, the in-vivo reference susceptibility value was chosen as the mean susceptibility of cerebrospinal fluid in the ventricles.

### Longitudinal assessment of human brain magnetic susceptibility measured ex-vivo

The longitudinal data from Dataset 1 were used to study the behavior of magnetic susceptibility in human brain hemispheres as a function of time postmortem. First, for each hemisphere, magnetic susceptibility maps from consecutive time-points were pair-wise spatially matched by non-linearly co-registering the corresponding spin-echo data (using DRAMMS [34]) and applying the resulting transformations to the susceptibility maps (since the brain hemispheres were not moved between sequences and the spin-echo and gradient-echo data shared the same space), and difference maps were generated for each pair of time-points. The difference maps were visually inspected for systematic changes in susceptibility over time postmortem. In addition, fifteen regions of interest located throughout the brain hemispheres were automatically selected and mean susceptibility values from these regions were recorded for each time-point. The selected regions of interest included the globus pallidus, putamen, caudate, six cortical gray matter regions and six white matter regions located in the anterior, posterior, medial, lateral, superior and inferior portions of the hemispheres. Region selection was accomplished by a) defining the regions of interest in a brain hemisphere template constructed using ex-vivo spin-echo data from 80 MAP/ROS participants, b) non-linearly registering the template to the spin-echo data from individual brain hemispheres collected at approximately six weeks postmortem, c) non-linearly registering the latter to the spin-echo data from all other time-points (DRAMMS [34]), and d) applying the combination of the resulting transformations to the regions of interest in template space to transform them to the space of the spin-echo data from different time-points with a single interpolation. Region positioning was corrected manually where necessary. The mean and 95% confidence interval of the susceptibility values in the selected regions of interest were extracted for each hemisphere and plotted as a function of time postmortem. Linear mixed modeling with random effects for the slope and intercept was used to assess the association of ex-vivo magnetic susceptibility measurements from all regions of all hemispheres of Dataset 1 with time postmortem. Significance was defined at p<0.05.

### Relationship between magnetic susceptibility measurements conducted in-vivo and ex-vivo

The in-vivo and ex-vivo data on the same 11 participants (Dataset 2) were used to study the relationship between in-vivo and ex-vivo magnetic susceptibility values. For each participant, single hemisphere image volumes were digitally extracted from the whole brain in-vivo gradient-echo data. The gray matter regions of interest discussed above were automatically selected in the in-vivo and ex-vivo data from the same participants (white matter regions were not considered in this portion of the analysis due to the different orientation of brain hemispheres during in-vivo and ex-vivo imaging, and the orientation dependence of magnetic susceptibility). To accomplish automated region selection, the brain hemisphere template discussed above was first non-linearly registered to the ex-vivo spin-echo data of Dataset 2, and ex-vivo gradient-echo data were non-linearly registered to the corresponding in-vivo data (using DRAMMS [34]). Since ex-vivo spin-echo and gradient-echo data shared the same space, the resulting transformations were appropriately combined and applied to the regions of interest in template space to transform them to the space of the in-vivo and ex-vivo gradient-echo data from individual hemispheres with a single interpolation. Region positioning was corrected manually where necessary. Regional in-vivo and ex-vivo mean susceptibility values were recorded for each hemisphere and were plotted against each other. Since the magnetic susceptibility of paramagnetic sources is temperature dependent, ex-vivo magnetic susceptibility values were temperature corrected to the in-vivo condition using Curie’s law (from 22.5°C to 36.5°C) [4]. Ex-vivo magnetic susceptibility measurements from all gray matter regions of interest were regressed on the corresponding in-vivo measurements using linear mixed modeling with random effects for the slope and intercept. The same process was repeated for 1000 bootstrapped copies of the eleven hemispheres, sampled with replacement. For each iteration, the ratio of the residual variance of the regression over the total variance of magnetic susceptibility measured ex-vivo was calculated. A low value for this ratio suggests that ex-vivo magnetic susceptibility values can be well modeled as a linear function of susceptibility measured in-vivo. The mean, standard deviation, and 95% confidence interval of this ratio and the slope of the model over all iterations, were calculated.

In order to investigate the effects of the differences between in-vivo and ex-vivo acquisition protocols and processing streams on the relationship between in-vivo and ex-vivo magnetic susceptibility measurements, one brain hemisphere was imaged ex-vivo using both protocols (Dataset 3) and the data were processed using the corresponding processing streams and evaluated. More specifically, the 3T and 1.5T gradient-echo data of Dataset 3 were co-registered using rigid body registration. The fifteen gray and white matter regions of interest discussed above were automatically selected in the 3T and 1.5T data, and mean susceptibility values were recorded for all regions. Regional 3T measurements were regressed on the corresponding 1.5T measurements using linear regression. The same process was repeated for 1000 bootstrapped copies of the 15 regions of interest, sampled with replacement. For each iteration, the ratio of the residual variance of the regression over the total variance of the 3T measurements was calculated. A low value for this ratio suggests that the susceptibility measurements obtained with the 3T acquisition protocol and corresponding processing stream can be well modeled as a linear function of the susceptibility measured with the 1.5T protocol and processing stream. The mean, standard deviation, and 95% confidence interval of this ratio and the slope of the model over all iterations, were calculated.

## Results

### Ex-vivo human brain magnetic susceptibility maps

**Fig 2:**
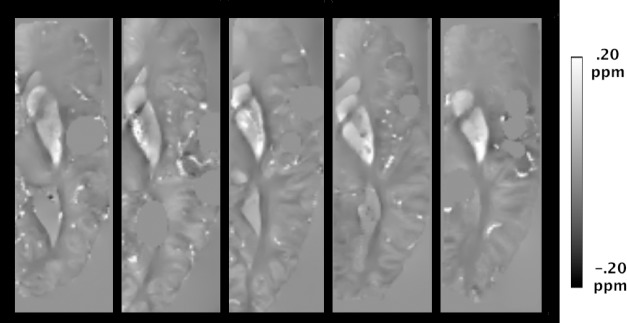
Ex-vivo magnetic susceptibility maps. Examples of axial ex-vivo magnetic susceptibility maps of human brain hemispheres from five participants.

Visual inspection of ex-vivo human brain magnetic susceptibility maps revealed features similar to those of published in-vivo maps on older adults (Fig 2) [6]. For example, high magnetic susceptibility was found in subcortical gray matter regions, specifically the putamen, globus pallidus, and caudate, which are known to be iron rich [6]. In addition, magnetic susceptibility was lower on average in white matter compared to gray matter, due to the diamagnetic nature of myelin [35]. Furthermore, magnetic susceptibility varied throughout white matter, probably due to the dependence of susceptibility on the orientation of axons, as well as due to differences in myelin content [36–38]. Lastly, gray-white matter contrast was stronger in the occipital lobes than the frontal lobes [39]. Despite the similarities between in-vivo and ex-vivo susceptibility maps, ex-vivo maps included signal voids near air pockets. Since, air trapped in the ventricles or on the surface of brain hemispheres can corrupt magnetic susceptibility calculations, regions near air pockets were masked out prior to removing the background field, and susceptibility calculations were not conducted in those regions, leaving visible signal voids (Fig 2).

### Longitudinal assessment of human brain magnetic susceptibility measured ex-vivo

**Fig 3:**
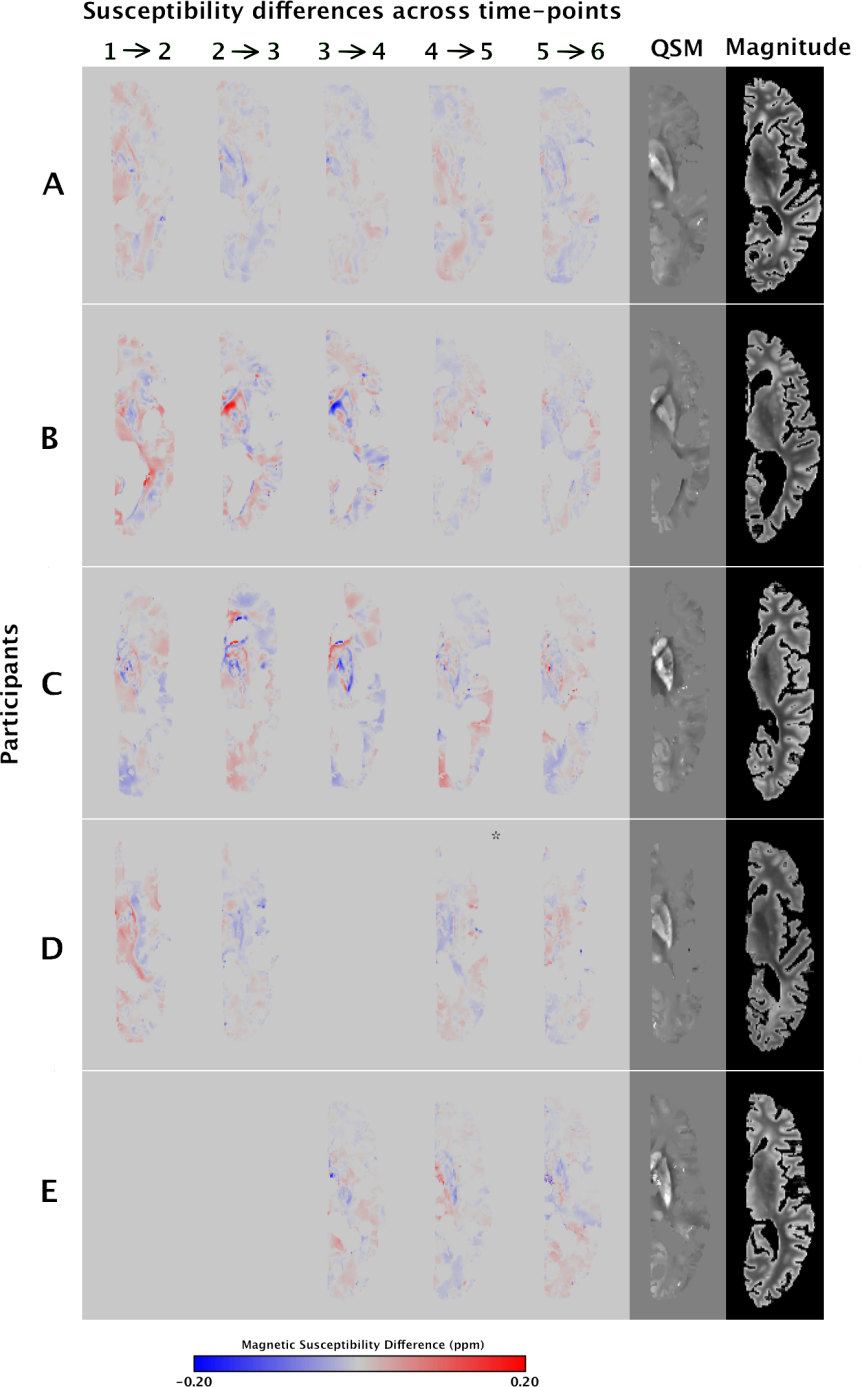
Voxel-wise differences in magnetic susceptibility maps of consecutive time-points. Ex-vivo magnetic susceptibility difference maps between consecutive time-points, for all hemispheres of Dataset 1. The corresponding susceptibility map and spin-echo image of the last time-point are displayed on the rightmost two columns. Note: For hemisphere D, the difference map marked with an asterisk represents the difference between the third and fifth time-points.

**Fig 4:**
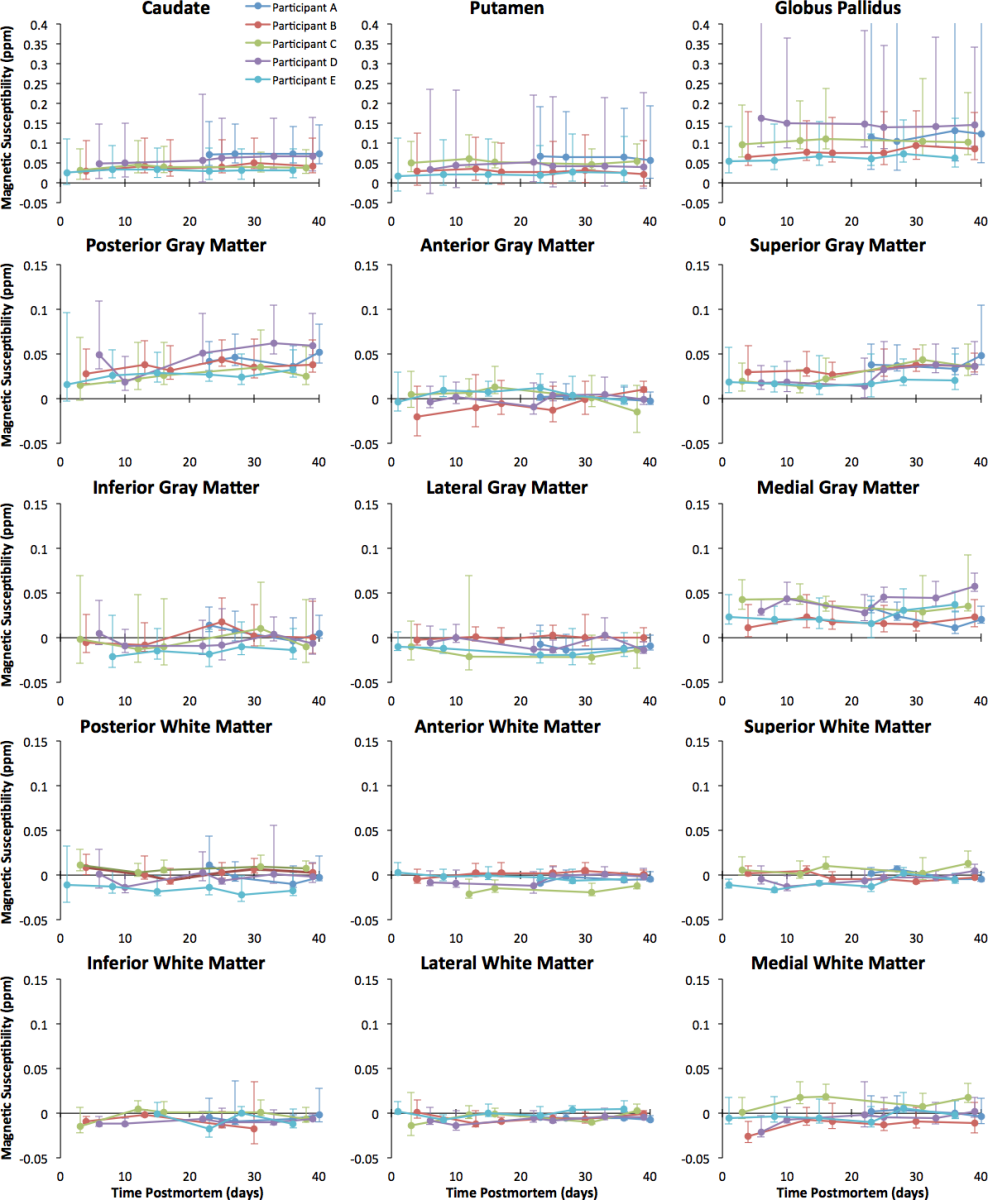
Magnetic susceptibility over time postmortem for gray and white matter regions. Plots of ex-vivo magnetic susceptibility in selected gray and white matter regions as a function of time postmortem, for all hemispheres of Dataset 1. Error bars around individual data points represent the 95% confidence interval of the susceptibility values within the region at that specific time point.

Ex-vivo magnetic susceptibility difference maps between consecutive time-points revealed no systematic susceptibility changes over time postmortem (from 0 days to 6 weeks postmortem) in the five participants of Dataset 1 (Fig 3). Also, linear mixed modeling yielded no significant association of ex-vivo magnetic susceptibility values in the gray and white matter regions of interest with time postmortem (p = 0.07) (Fig 4). Magnetic susceptibility in subcortical gray matter regions (caudate, putamen, and globus pallidus) exhibited higher variation across participants than in white matter and cortical gray matter regions (Fig 4).

### Relationship between magnetic susceptibility measurements conducted in-vivo and ex-vivo

**Fig 5:**
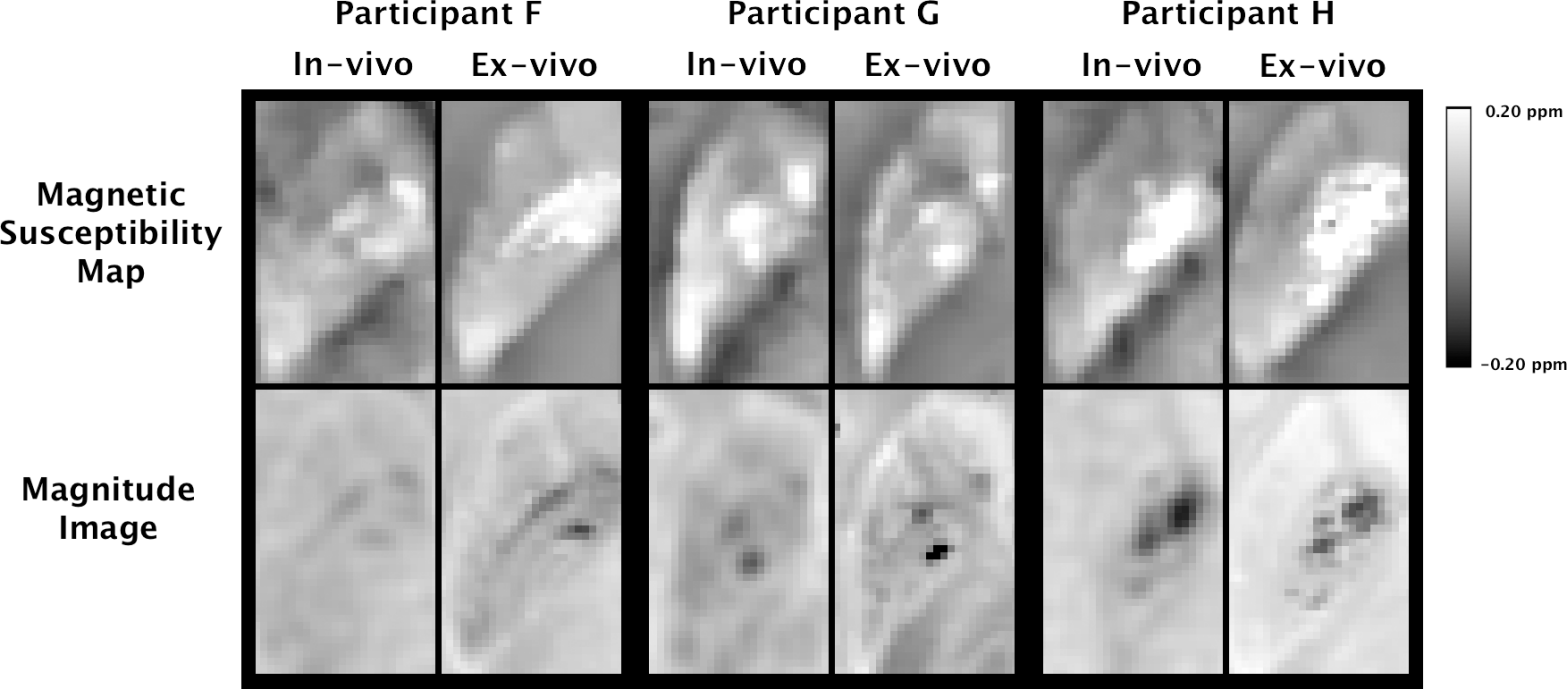
Correspondence of in-vivo and ex-vivo data within the same participants. In-vivo and ex-vivo magnetic susceptibility and gradient-echo magnitude maps for a section of the basal ganglia of three hemispheres from Dataset 2 imaged both in-vivo and ex-vivo.

**Fig 6:**
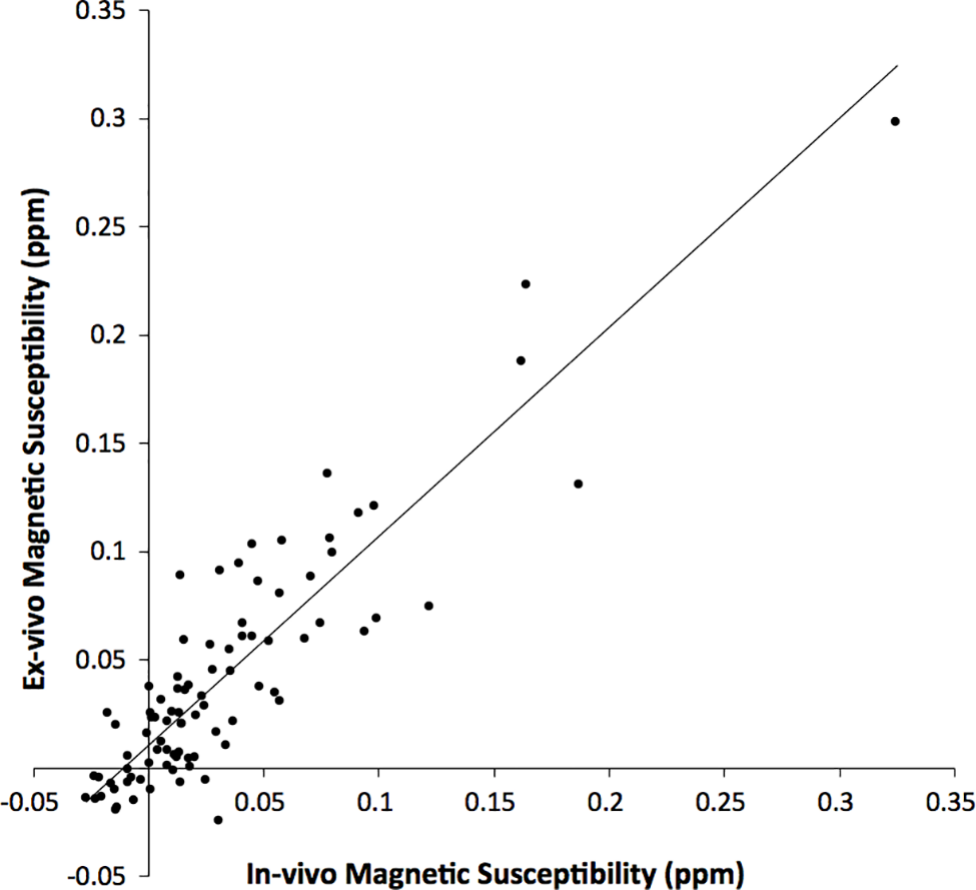
Ex-vivo magnetic susceptibility as a function of in-vivo magnetic susceptibility. Plot of regional gray matter magnetic susceptibility values measured ex-vivo as a function of the corresponding susceptibility values measured in-vivo in the same hemispheres, for all hemispheres of Dataset 2. Each point in the scatter plot represents a single gray matter brain region of a single hemisphere. Ex-vivo magnetic susceptibility values shown in the plot have been corrected for the effects of lower temperature during ex-vivo imaging [4].

Visual inspection showed good agreement between in-vivo and ex-vivo magnetic susceptibility maps from the same hemispheres, in terms of signal patterns (Fig 5). The ratio of the residual variance in the regression of regional ex-vivo magnetic susceptibility values on the corresponding in-vivo measurements from the same participants over the total variance of magnetic susceptibility measured ex-vivo was relatively small at 0.19 ± 0.06 (95% confidence interval of [0.11, 0.33]). This suggests that magnetic susceptibility measured ex-vivo may be well modeled as a linear function of magnetic susceptibility measured in-vivo (Fig 6). The mean slope of the bootstrapped linear mixed model was 1.03 ± 0.07 (95% confidence interval of [0.89, 1.17]) (Fig 6).

**Fig 7:**
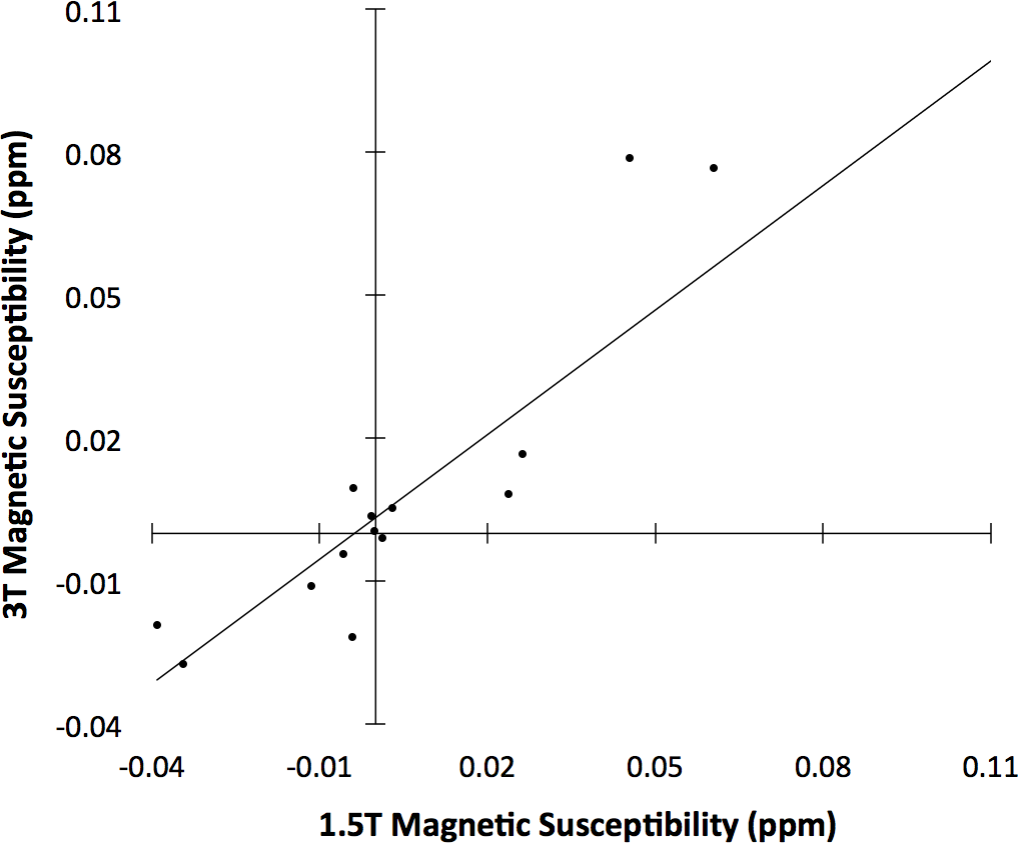
Measurements from 3T as a function of measurements from 1.5T. Plot of magnetic susceptibility in selected gray and white matter regions of the same hemisphere imaged postmortem using both in-vivo (1.5T) and ex-vivo (3T) QSM methods (Dataset 3).

The investigation on the effects of the two sets of acquisition protocols and processing streams used on the in-vivo and ex-vivo data of Dataset 2 demonstrated that, magnetic susceptibility measurements based on the 3T acquisition and processing methods were well modeled as a linear function of susceptibility measurements based on the 1.5T methods in the same hemisphere. More specifically, the ratio of the residual variance of the regression of regional 3T measurements on the corresponding 1.5T measurements over the total variance of 3T measurements was relatively small at 0.15 ± 0.08 (95% confidence interval of [0.03, 0.34]). This suggests that the susceptibility measurements obtained with the 3T acquisition protocol and corresponding processing stream may be well modeled as a linear function of the susceptibility measured with the 1.5T protocol and processing stream (Fig 7). The mean slope of the bootstrapped linear model was 0.91 ± 0.17 (95% confidence interval of [0.59, 1.29]) (Fig 7).

## Discussion

Ex-vivo QSM allows investigation of brain characteristics at essentially the same point in time as histopathologic examination, independent of frailty level, and therefore has the potential to become an important tool for the assessment of the neuropathologic correlates of magnetic susceptibility in aging. In the present study, magnetic susceptibility in gray and white matter of five human brain hemispheres was monitored over time postmortem, and no systematic change was observed in the first six weeks after death. This information is important for future cross-sectional ex-vivo QSM studies of hemispheres imaged at different postmortem intervals. Additionally, a close linear relationship was detected between in-vivo and ex-vivo gray matter magnetic susceptibility measurements from eleven participants, suggesting that ex-vivo QSM captures information linked to antemortem gray matter magnetic susceptibility.

To our knowledge, this is the first longitudinal ex-vivo QSM investigation of the human brain. Nevertheless, the novel finding that magnetic susceptibility in gray and white matter does not significantly change with time postmortem for the first six weeks after death is in general agreement with studies on the effects of fixation on major determinants of magnetic susceptibility in the brain, such as iron and myelin, among others. Previous work examining the effects of fixation on the concentration of iron [4] showed that, in deep gray matter of cadaveric human brains, iron concentration was unaffected by fixation for a storage time of less than one year [40, 41]. In addition, analysis of the fixation solution used for different human brains showed no significant leaching of iron until long term storage (>20 years) [40]. Furthermore, no significant differences in deep gray matter iron concentrations were detected between frozen and fixed brains [41], and the relationship between magnetic susceptibility and iron concentration was similar in both fixed and unfixed cadaveric brains [4, 5]. In terms of myelin, a previous study showed that white matter components, including myelin, were well preserved with fixation [42]. This postmortem temporal stability of major determinants of magnetic susceptibility in the human brain implies potentially stable susceptibility values over time postmortem. The present study provides the first direct evidence on the temporal stability of magnetic susceptibility in gray and white matter of human brain hemispheres for the first six weeks postmortem. This information is crucial for future cross-sectional ex-vivo QSM studies of human brain hemispheres that may be imaged at different postmortem intervals [43].

Gray matter magnetic susceptibility measured ex-vivo was linearly related to magnetic susceptibility measured in-vivo in the same participants. This suggests that ex-vivo QSM captures information linked to antemortem gray matter magnetic susceptibility, which is essential for translating ex-vivo QSM findings to in-vivo. Also, considering the advantages of combining ex-vivo MRI (in contrast to in-vivo MRI) with histopathologic examination, this study indicates that ex-vivo QSM may become an important tool for the assessment of the neuropathologic correlates of magnetic susceptibility in aging.

The value of the slope of the linear relationship between ex-vivo and in-vivo gray matter magnetic susceptibility measurements was approximately equal to one. This suggests a straightforward translation of ex-vivo susceptibility values to in-vivo. However, a number of factors influenced this slope, and it is therefore important to discuss them here. Firstly, there were differences in QSM data acquisition methods used in-vivo and ex-vivo. Although susceptibility values have been shown to be consistent across magnetic field strengths [44], they may have been underestimated in-vivo due to the lower spatial resolution of the in-vivo compared to the ex-vivo acquisitions [45, 46], thereby increasing the slope of the linear relationship between ex-vivo and in-vivo gray matter. Differences in QSM data acquisition methods led to differences in post-processing methods, and the effects of the latter on susceptibility estimation have not yet been described in the literature. Furthermore, changes in total blood volume per voxel, chemical, physiological and structural changes that occur at death and after extraction from the skull may have also influenced the value of the slope of the linear relationship between ex-vivo and in-vivo susceptibility measurements. Finally, in-vivo QSM was conducted 0.3 to 3.6 years prior to death, which although is a relatively short period of time compared to the literature, it may have allowed additional pathology to develop after in-vivo imaging and prior to autopsy, potentially introducing differences in magnetic susceptibility between the in-vivo and ex-vivo data. Nevertheless, independent of the exact value of the slope, the linear relationship between ex-vivo and in-vivo magnetic susceptibility measurements is sufficient for purposes of translation of ex-vivo findings, as discussed above, and is therefore of high significance.

Magnetic susceptibility in subcortical gray matter regions exhibited higher variation across participants than in cortical gray matter and white matter regions. It is well known that aging is associated with increased mineralization of subcortical gray matter [47–49]. Differences in age-related neuropathology burden across the older adults participating in this study may have led to different levels of mineralization of subcortical gray matter, which may be responsible for the higher variance in magnetic susceptibility in that portion of the brain. The exact links between mineralization and different age-related neuropathologies are not yet clear. The present work will actually serve as the foundation for future ex-vivo QSM-pathology studies that will reveal the links between magnetic susceptibility and age-related neuropathologies.

This study also has limitations. First, the orientation of brain hemispheres with respect to the main magnetic field was different in-vivo and ex-vivo, because it is difficult to achieve supine-like positioning for a brain hemisphere that is immersed in fixative. Since magnetic susceptibility depends on the orientation of axons, white matter susceptibility measurements were not used when studying the relationship between in-vivo and ex-vivo magnetic susceptibility. White matter susceptibility measurements were included in the longitudinal assessment of susceptibility measured ex-vivo. Nevertheless, we anticipate a similar linear relationship between in-vivo and ex-vivo white matter magnetic susceptibility measurements as in gray matter. Second, residual misregistration across time-points postmortem or between in-vivo and ex-vivo datasets, may have introduced noise in susceptibility measurements in the regions of interest. Despite the above limitations, this work generated results that lay the foundation for future studies of aging involving ex-vivo QSM.

## Conclusion

The present study aimed at assessing the behavior of magnetic susceptibility in gray and white matter of human brain hemispheres as a function of time postmortem, and establishing the relationship between gray matter susceptibility measurements conducted in-vivo and ex-vivo on the same brain hemispheres. It was demonstrated that, for the experimental procedures used in this work, gray and white matter magnetic susceptibility did not systematically change in the first six weeks after death. This finding suggests that future cross-sectional ex-vivo QSM studies may not be contaminated by differences in postmortem interval to imaging across participants, as long as that interval is less than six weeks. Additionally, it was shown that magnetic susceptibility of gray matter measured ex-vivo may be well modeled as a linear function of magnetic susceptibility measured in-vivo, suggesting that ex-vivo QSM captures information linked to the antemortem magnetic susceptibility of gray matter in the human brain.
